# Ion therapy of prostate cancer: daily rectal dose reduction by application of spacer gel

**DOI:** 10.1186/s13014-015-0348-1

**Published:** 2015-02-27

**Authors:** Antoni Rucinski, Stephan Brons, Daniel Richter, Gregor Habl, Jürgen Debus, Christoph Bert, Thomas Haberer, Oliver Jäkel

**Affiliations:** Heidelberg Ion-Beam Therapy Center (HIT) and Department of Radiation Oncology, University Clinic Heidelberg, Im Neuenheimer Feld 400, 69120 Heidelberg, Germany; Biophysics Division, GSI Helmholtzzentrum für Schwerionenforschung GmbH, Planckstraße 1, Darmstadt, Germany; Deutsches Krebsforschungszentrum, Im Neuenheimer Feld 280, 69120 Heidelberg, Germany; Now INFN Sezione di Roma, Roma, Italy and Dipartimento di Scienze di Base e Applicate per Ingegneria, Sapienza Universit’a di Roma, Roma, Italy; Now Friedrich-Alexander Universität Erlangen-Nürnberg and University Clinic Erlangen, Radiation Oncology, Universitätsstraße 27, 91054 Erlangen, Germany; Now Department of Radiation Oncology, Technische Universität München (TUM), Munich, Germany

**Keywords:** Prostate cancer, Ion therapy, Proton therapy, Spacer gel, Treatment planning

## Abstract

**Background:**

Ion beam therapy represents a promising approach to treat prostate cancer, mainly due to its high conformity and radiobiological effectiveness. However, the presence of prostate motion, patient positioning and range uncertainties may deteriorate target dose and increase exposure of organs at risk. Spacer gel injected between prostate and rectum may increase the safety of prostate cancer (PC) radiation therapy by separating the rectum from the target dose field. The dosimetric impact of the application of spacer gel for scanned carbon ion therapy of PC has been analyzed at Heidelberg Ion-Beam Therapy Center (HIT).

**Materials and methods:**

The robustness of ion therapy treatment plans was investigated by comparison of two data sets of patients treated with and without spacer gel. A research treatment planning system for ion therapy was used for treatment plan optimization and calculation of daily dose distributions on 2 to 9 Computed Tomography (CT) studies available for each of the 19 patients. Planning and daily dose distributions were analyzed with respect to target coverage, maximal dose to the rectum (excluding 1 ml of the greatest dose; Dmax-1 ml) and the rectal volume receiving dose greater than 90% of prescribed target dose (V90_Rectum_), respectively.

**Results:**

The application of spacer gel did substantially diminish rectum dose. Dmax-1 ml on the treatment planning CT was on average reduced from 100.0 ± 1.0% to 90.2 ± 4.8%, when spacer gel was applied. The robustness analysis performed with daily CT studies demonstrated for all analyzed patient cases that application of spacer gel results in a decrease of the daily V90_Rectum_ index, which calculated over all patient cases and CT studies was 10.2 ± 10.4 [ml] and 1.1 ± 2.1 [ml] for patients without and with spacer gel, respectively.

**Conclusions:**

The dosimetric benefit of increasing the distance between prostate and rectum using spacer gel for PC treatment with carbon ion beams has been quantified. Application of spacer gel substantially reduced rectal exposure to high treatment dose and, therefore, can reduce the hazard of rectal toxicity in ion beam therapy of PC. The results of this study enable modifications of the PC ion therapy protocol such as dose escalation or hypofractionation.

**Electronic supplementary material:**

The online version of this article (doi:10.1186/s13014-015-0348-1) contains supplementary material, which is available to authorized users.

## Introduction

In the last decade radiotherapy became one of the most often applied prostate cancer (PC) treatment methods. Several studies demonstrated that increased Tumour Control Probability (TCP) of localized PC can be achieved by dose escalation and application of hypo-fractionation treatment protocol [1-7]. However, the target dose is constrained by dose limits to the surrounding organs at risk: rectum and bladder. In addition, inter- and intrafractional anatomy variations in the target region increase the hazard of underdosage of prostate and overdosage of surrounding tissue, which might result in toxicity of organs at risk. Increasing target dose is possible by application of motion mitigation techniques or/and by improvements of irradiation accuracy realized, among others, by application of conformal treatment techniques, such as Intensity Modulated Radiation Therapy (IMRT), Image Guided Radiation Therapy (IGRT) or arc-based therapy techniques.

In this context, ion beam therapy offers a possibility of further hypo-fractionation by the PC radiotherapy, mainly due to the sharper than in photon therapy dose gradients achieved thanks to the characteristic physical properties of ions (Bragg peak) [8]. Especially the irradiation of PC with carbon ion beams is expected to provide higher treatment efficacy [9,10], since carbons demonstrate increased radio-biological effectiveness in comparison with photon or proton irradiation. However, the high conformity of ion therapy using raster scan technique is challenged by organ motion that may strongly affect the quality of target irradiation [11]. Due to the finite range of the ions in tissue, deviations in the target dose may occur as a result of variations of material density distribution over the particle path mainly induced by daily target motion and patient positioning [12].

The challenge of hypo-fractionated prostate treatment with scanned ion beams is to limit the hazard of motion induced dose to the most critical organ at risk, rectum. Currently, in ion therapy, patient positioning is typically performed by matching of bony anatomy on the co-planar radiography images [13]. This patient positioning procedure does not allow to image prostate, rectum and bladder (no soft tissue contrast) and the exact location of these organs in relation to femur bones cannot be controlled on a daily basis. This study confirms findings made in photon therapy [14], which show that because of different rectum or bladder filling than by treatment planning imaging, rectum might be exposed in a single treatment fraction to the dose substantially higher than predicted by treatment planning. This situation cannot be diagnosed without advanced in-room IGRT methods like tomography imaging offering soft tissue contrast or radiography imaging applied in combination with injection of radio-opaque markers to prostate. Even if application of in-room tomography imaging would be a standard (in-room Computed Tomography - CT, Magneto Resonance Imaging - MRI), the admissible fraction and total radiobiological dose limit to the rectum for carbon ion therapy is an open discussion topic and robustness analysis including radiobiological effects of ion radiation as well as clinical experience is required [7,10].

For these reasons, the application of hydro-gel distancing prostate and rectum allows also mitigating the problems described above and decreasing the potential risk of rectal toxicity by ion beam irradiation of PC. Several studies quantified dosimetric benefit to rectum due to the application of spacer gel in photon therapy, possibly allowing higher doses for target [15,16]. First annotation considering application of spacer gel for proton therapy and its dosimetric effects was published by Weber [17]. Our previous preclinical investigations considered the impact of spacer gel application on treatment planning (TP) with ion beams and its potential application for in-vivo range verification [18]. In that work we demonstrated that spacer gels do not change their physical properties under exposure to high doses of ion irradiation and the penetration depth of ion beams in gel is stable over therapy course and could be properly predicted by the CT imaging. On the basis of these findings safety of treatment planning for ion therapy of PC is guaranteed, which allows reliable dose distribution calculations necessary for the robustness analysis presented in this work.

Further, fundamentally different approach of application of spacer gel in combination with ion beam therapy was proposed by Christodouleas et al. [19], who simulated application of anterior proton beams, as an alternative to conventional, opposing horizontal beams protocol, typically applied in the proton therapy. Christodouleas et al. suggests using spacer gel to spare rectum from the effects of possible range uncertainties and dose deposition to the rectum, which are probable by anterior irradiation due to the varying bladder filling. Approach proposed by Christodouleas et al. is conditioned by availability of range verification methods in the clinical routine.

The goal of presented TP study was to investigate the dosimetric impact of spacer gel application on safety of daily irradiation of PC with horizontal ion beams in presence of motion. Treatment plans and daily dose recalculations performed on CT studies of prostate patients with and without spacer gel implant were analyzed in order to investigate robustness of ion beam irradiation when spacer is applied. This study was a complementary investigation to Ion Prostate Irradiation (IPI) trial [10] performed at Heidelberg Ion Beam Therapy Centre (HIT), Heidelberg, Germany and used its clinically applied TP protocols.

## Materials and methods

### Patient data

Computed Tomography (CT) studies of patients treated with photons and ions in Heidelberg were used for the retrospective analysis. The studies obtained for TP as well as verification purposes were used for each patient. In total, 88 CT studies of 9 patients (59 CT studies) treated in years 2005–2006 in German Cancer Research Center (DKFZ), Heidelberg, Germany and 10 patients (29 CT studies) treated at HIT in 2012 were analyzed (Table 1). Patients treated at DKFZ who did not have spacer gel implant are called in this study *No-Spacer-Data* and are labeled patient *#1-#9*. Patients treated at HIT who had spacer gel implant are called in this study *Spacer-Data* and are labeled patient *#10-#19* (Table 1). The parameters of the CT scans used to obtain the patient data are specified in Table 2. In the presented retrospective analysis the variable number of CT studies between *Spacer-Data* and *No-Spacer-Data* has its reason in a different purpose of the CT acquisitions. *No-Spacer-Data* were obtained for weekly patient position control [20]. The *Spacer-Data* were obtained 1–2 times during the therapy course (apart of one exception, patient #12) with a research purpose of post treatment ion beam range verification [21].

**Table 1:** **This table provides number of CT studies including number of slices available per patient for** ***No-Spacer-Data*** **(patients from DKFZ) and** ***Spacer-Data*** **(patients from HIT)**

**A**	***No-Spacer-data***									***Spacer-data***									
B	#1	#2	#3	#4	#5	#6	#7	#8	#9	#10	#11	#12	#13	#14	#15	#16	#17	#18	#19
C	7	8	5	6	9	6	6	6	6	2	3	8	2	2	2	2	2	3	3
D	**68**	**65**	**67**	**67**	**36**	**67**	**67**	**67**	**63**	**168**	**446**	**557**	**175**	**156**	**182**	**155**	**156**	**500**	**162**
	68	67	67	67	37	67	67	67	67	75	150	225	75	75	75	75	75	225	75
	68	67	68	67	36	67	67	67	47		150	225						225	75
	67	67	67	67	60	67	67	67	47			225							
	67	67	67	67	67	67	67	67	47			225							
	67	67		67	36	67	67	67	47			225							
	67	65			49							225							
		60			49							225							
					49														

A - Data set description, B – Patient number, C- Number of CT studies including TP-CT, D – number of CT slices per CT study (TP CT is bold).

**Table 2:** **Scan parameters used for CT data acquisition of prostate patients**

	**Spacer-data**		**No-Spacer-data**
	**Sensation 4**	**Biograph mCT**	**Emotions**
Integrated current [mAs]	240	255	72-82
Tube output voltage [kV]	120*		110-130
Reconstruction diameter [mm]		500**	
Pixel size [mm]		0.9765 × 0.9765**	
Slice distance [mm]		3**	
Transversal CT grid [pixels]		512×512**	

Filtered back-projection reconstruction algorithm was used for all reconstructions. *The same number for two columns. **The same number for three columns.

### No-spacer-data

A Single-slice spiral CT scanner Siemens Emotion is located in the photon therapy treatment room of DKFZ in Heidelberg. This “on-rail” CT unit is set 90° apart from the 6 MV Siemens Primus linear accelerator (Siemens OCS, Concorde, CA) and is used for daily pre-treatment patient positioning [20]. The therapy couch is shared by both devices. CT scans were performed in a treatment position. The scan slices containing target volume plus at least 2 cm in cranial and caudal direction were obtained. Prostate patients treated in DKFZ were immobilized using stereotactic frames (wrap-around body cast and a head mask), which were removed from the images by setting the Hounsfield Unit (HU) values around the contour of patient skin to the air HU value (−1024 HU). Original treatment plans optimized for photons were not used in the presented analysis in order to avoid the HU uncertainties related to performing dose recalculation on the CT studies obtained with a different CT scanner than one used for TP. For the study purposes, new ion therapy treatment plans were optimized on the basis of obtained daily CT studies. Physician selected CT study for TP according to applied in the clinic procedure which require patient coming for CT imaging as well as during all therapy fractions with empty rectum and full bladder. Rectum diameter of about 4 cm on the tranversal view on the CT image in the PTV region was considered as appropriate to select CT study for TP.

HU stability of this data set was proved by evaluation of the HU values distribution within the Regions of Interest (ROI) delineated for TP on the clinical patient data. The admitted variation of average HU value within a ROI on daily CT study in comparison to the selected reference TP CT was checked to be within limits allowed by Quality Assurance (QA) procedure used at HIT. Details of HU analysis performed for *No-Spacer-Data* are presented in the “Additional file 1”.

### Spacer-data

Prostate patients treated at HIT, typically, at least one day prior to CT imaging, got spacer gel (SpaceOAR™ System, Augmenix Inc., Waltham, MA, US) implanted in the urology department of University Clinic Heidelberg. Spacer gel precursors are injected under ultrasound guidance into potential space between Dennonvilliers’ Fascia and the frontal rectal wall and polymerize (solidify) within seconds. The additional space created between prostate and rectum has a volume of about 10–15 ml. More specific information about application of spacer gel for ion beam therapy could be found in our previous publication [18]. Prostate patients treated at HIT were immobilized using ProStep™ System (Elekta AB (Publ), Stockholm, Sweden).

CT sudies dedicated for TP at HIT were obtained with SIEMENS *Sensation 4* installed in the University Clinic in Heidelberg. The control CT studies were obtained using Positron Emission Tomography/Computed Tomography (PET/CT) unit SIEMENS *Biograph mCT* installed next door to the ion therapy treatment room. At HIT PET/CT is dedicated to post-therapeutic PET measurements performed for in-vivo treatment monitoring [21]. Prostate patients after the therapy course were either shuttled in the treatment position to the PET room or (having visited the restroom) were re-positioned on the couch of the PET/CT device (approximately 15 minutes after the end of therapy). CT studies obtained for attenuation correction of PET acquisitions were used for the analysis presented in this study. The CT unit of the PET/CT device is commissioned for TP, which allows a reliable dose distribution calculation of treatment plans optimized for the therapy and applied to the patients at HIT. The stability of the HU values between TP CT and daily CT images is guaranteed by the periodic QA procedures.

### Registration of CT studies

HIT offers digital X-ray based patient position verification system for ion beam therapy of prostate patients. The radiography based image guidance does not provide the information about dosimetric effect of soft tissue variations (including influence of spacer gel) and range uncertainties which are the focus of presented investigation. In order to concentrate on the soft tissue displacements, maintaining the registration procedure most accurate and excluding positioning uncertainties from the dosimetric analysis, an automated bony anatomy-based CT study registration including translations and rotations was performed using Siemens SyngoRT planning software package (version VA11A). This procedure imitated ideal ion therapy treatment conditions possible to achieve with cone beam CT based positioning allowing 3D imaging with soft tissue contrast. A fine image alignment was additionally performed by a physician, if a rotation of femur bones occurred in comparison to the TP CT.

For the Spacer-Data, CT studies acquired with the CT unit of the PET/CT device were registered to the TP CT studies. For No-Spacer-Data, for each patient physician selected a representative daily CT from entire data set as TP CT and remaining images were registered to the selected one. The registered images were re-sampled to the dimensions of the planning CT cube.

For both data sets different, highly precise immobilization methods were applied: wrap-around body cast and a head mask for No-Spacer-Data set and pro-step system for Spacer-Data set. In this work it was assumed that independent on patient immobilization method, possible positioning inaccuracies could be neglected in the dosimetric comparison of data sets with and without spacer gel, if rigid registration of CT studies based on femur bones matching is applied.

### Contour segmentation

The original contours segmented on the TP CT studies used for TP at HIT were adopted for this work. On TP CT and daily CT studies of *No-Spacer-Data* set as well as on daily CT studies of *Spacer-Data* set new contours were segmented according to the clinical protocol of the IPI study.

All contours were segmented manually on the transversal view of TP CT for each slice individually. Organs at risk: bladder, rectum and femur bones were contoured by a medical physicist and controlled and corrected by physician. Target structures: GTV (prostate), Clinical Target Volume (CTV), Planning Target Volume (PTV), and contour of spacer gel (Gel) were delineated by the physician who additionally used soft tissue information from fused MRI. According to the protocol of IPI study CTV was defined as prostate gland (GTV) + 2 mm including 2/3 of Seminal Vesicles. PTV was an anisotropic margin extension around CTV: 5 mm in anterior-posterior (AP) and superior-inferior (SI) direction and 7 mm in left-right (LR) direction in CT coordinate system. Margin extension in the AP and SI direction guarantees CTV coverage in presence of prostate motion during the therapy. Since ion therapy of PC is realized at HIT by application of two opposing horizontal 90° fields, margins extension in the LR direction guarantees CTV coverage in presence of range uncertainties caused by the inaccurate patient positioning.

### Treatment plan optimization

In this work, TRiP98 (TReatment planning for Particles) software package [22-24], developed at GSI for patient treatment within ion therapy pilot project, was used for optimization and dose distribution calculations. The physical beam-model, input data used by TRiP98 for the TP optimization and dose distribution calculation were the same as the data used by Siemens Syngo RT, software clinically used for the TP at HIT [25]. The biological optimization and dose distribution calculation for carbon ions were based on the biological model LEM I which was introduced by Scholz et al. [26,27] and is currently used for therapy at HIT. The biological optimization with ^12^C was performed with 3.3 Gy (RBE) target dose prescribed to the PTV, two opposing horizontal fields (90°), 3×3 mm lateral scanning grid, 3 mm water-equivalent iso-energy slice spacing and multi-field optimization algorithm working on CT-grid. The original treatment plans applied for irradiation of the patient at HIT were not used in this study.

For each patient of *Spacer-Data* set the TP optimization was performed with original CT studies clinically used at HIT with a target point defined manually by medical physicist. For each patient of *No-Spacer-Data* set one of daily CT studies was selected by physician for treatment plan optimization and target point was defined automatically by TRiP98 as the centre of mass of PTV volume.

### Dose calculation and data analysis

The robustness studies included application of the treatment plans optimized with TRiP98 to the daily CT studies. The target point and irradiation field previously used for TP optimization were also used for dose calculations.

**Figure 1:**
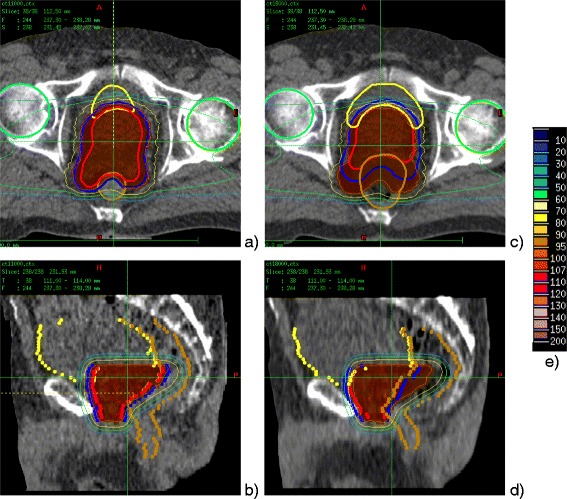
**Patient** ***#2*** **of** ***No-Spacer-Data*** **set: comparison of the dose distribution calculated for the “best case”, TP CT (left: a,b) and the “worst case” daily CT (right: c,d).** The figure shows transversal **(a,c)** and saggital **(b,d)** views. CT studies are overlapped with the segmented contours and dose distributions. Contours of the target (PTV – blue, CTV - red) and the organs at risk (rectum – brown, bladder - yellow) are delineated with the thick lines on the transversal view and dots on the saggital view. Dose distributions (dose legend - **e)** are presented with thin isodose lines of 30%, 50%, 70%, 95%. Color field was used for the highest dose regions of 95-100% and 100-107%.

**Figure 2:**
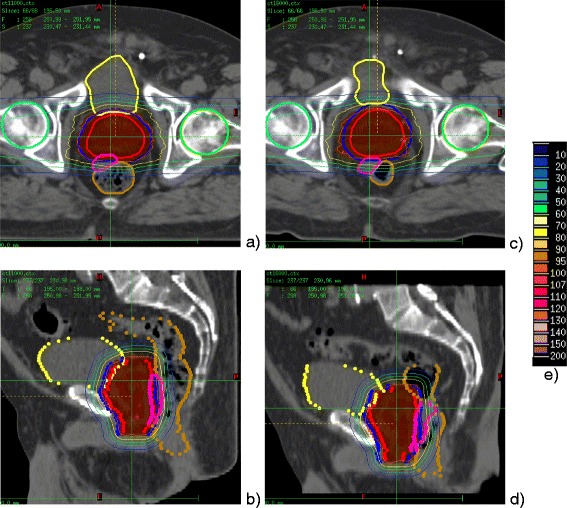
**Patient** ***#10*** **of** ***Spacer-Data*** **set: comparison of the dose distribution calculated for the “best case”, TP CT (left: a,b) and the “worst case” daily CT (right: c,d).** The figure shows transversal **(a,c)** and saggital **(b,d)** views. CT studies are overlapped with the segmented contours and dose distributions. Contours of the target (PTV – blue, CTV - red) and the organs at risk (rectum – brown, bladder - yellow) are delineated with the thick lines on the transversal view and dots on the saggital view. In addition, the contour of the spacer gel is delineated in pink. Dose distributions (dose legend - **e)** are presented with thin isodose lines of 30%, 50%, 70%, 95%. Color field was used for the highest dose regions of 95-100% and 100-107%. The comparison of the subfigures described with the same letters on the Figures 1 and 2 allow seeing the differences resulting from application of spacer gel.

In this study the dosimetric impact of application of spacer gel on rectal dose in presence of daily anatomy variations was evaluated by a comparison of *Spacer-Data* and *No-Spacer-Data* set. The three-dimensional (3D) dose distributions were quantitatively analyzed using TP parameters extracted on the basis of calculated dose distributions and the available contours. The TP parameters calculated from dose volume histograms (DVH) were used for evaluation according to the protocol of IPI study. If not specified differently, the values of TP parameters were presented using the following convention: median value (MD) ± standard deviation (STD). In addition to the quantitative analysis one patient of *Spacer-Data* and *No-Spacer-Data* set were selected as the example patients (Figures 1 and 2).

First, in order to assess the quality of the optimized TP, the dose distribution calculated for TP CT was analyzed. The PTV volume receiving the dose higher than 95% of the prescribed target dose (V95_PTV_), and dose conformity index defined as ratio of volume of the target and the volume of the 90% isodose (considered within and out of the target; CI90_PTV_) were evaluated to guarantee comparable quality of optimization for all investigated patients. The impact of application of spacer gel on the quality of TP optimization was evaluated by checking the maximal dose in clinically relevant rectal volume (more than 1 ml, Dmax-1ml_Rectum_). Second, from the daily dose distributions the daily coverage of the clinical target volume (V95_CTV_) and the daily rectal exposure (V90_Rectum_) were evaluated as a measure of impact of application of spacer gel on the patient dosimetry in presence of daily anatomy variations. The daily distribution of V90_Rectum_ index was illustrated for each patient using box plots. Red line is the median value of the distribution, the box extends to 50% of the data distribution (between the first and the third quartile), whiskers extend to the most extreme data point within 75% data range, the remaining data were marked as outliers with “+”.

## Results

In this study, the dosimetric impact of application of spacer gel in ion therapy was simulated and quantitatively evaluated in robustness analysis by analyzing daily dose distributions calculated for TP CT and daily CT studies of *Spacer-Data* and *No-Spacer-Data* set.

The treatment plans were optimized aiming to keep coverage of the PTV on the treatment plan similarly for each patient case. The analyzed data show that MD and STD of V95_PTV_ index and coverage index (CI90_PTV_) calculated on TP CT studies over both data sets are of 96.5 ± 0.6% and 86.5 ± 1.6%, respectively. The V95_PTV_ index greater than 95% fulfils the optimization condition. The low values of STD for V95_PTV_ and CI90_PTV_ demonstrate that the dose distribution over the target was optimized for both data sets in the comparable manner, which is required for reliable robustness analysis. The comparison of the dose distributions of the treatment plans (calculated on the TP CT) for patients with and without spacer gel shows that the application of spacer gel reduces the maximal dose to rectum (Dmax-1ml_Rectum_) from 100.0 ± 1.0% to 90.2 ± 4.8%. Further, the rectal volume receiving the high dose, greater than 90% of prescribe target dose (V90_Rectum_), decreases on the treatment plan from 5.9 ± 2.6 ml to 1.0 ± 1.1 ml when spacer gel is applied. The rectal volume receiving dose greater than 70% of prescribe target dose (V70_Rectum_) decreases from 12.2 ± 4.7 ml to 7.1 ± 2.2 ml due to the application of spacer gel prior the therapy.

**Figure 3:**
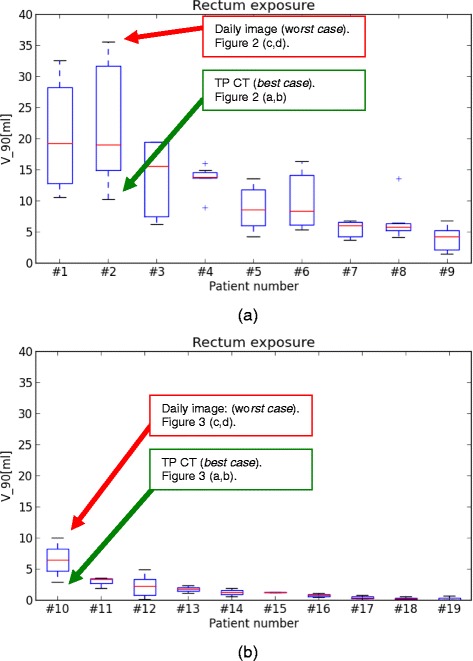
**Box plots showing the distribution of V90**_**Rectum**_ **index for patients without spacer gel implant (a) and with spacer gel implant (b).** Each box plots corresponds to the single patient case. Green and red arrow indicates the values of V90_Rectum_ index of these patients, who were selected to be illustrated as the patient examples on the Figures 1 and 2.

The robustness analysis performed in this work shows that application of spacer gel results in decrease of daily value of V90_Rectum_ index and, therefore, allows an improvement of rectum sparing over the whole therapy course for all analyzed patient cases. Figure 3a and 3b compare the results of the robustness analysis by means of distribution of V90_Rectum_ index extracted from daily 3D dose distributions for *No-Spacer-Data* and *Spacer-Data* set. For each patient the lowest value of V90_Rectum_ index corresponds to the rectal exposure on the TP CT study. The patients are sorted from the greatest median V90_Rectum_ index value to the lowest. For the *No-Spacer-Data* the maximal median rectal exposure was calculated for patient *#1*: V90_Rectum_ = 19.2 ± 18.8[ml]. For *Spacer-Data* set the maximal median rectal exposure was calculated for patient *#10*: V90_Rectum_ = 6.5 ± 5.1[ml]. V90_Rectum_ index calculated over all cases is V90_Rectum_ = 10.2 ± 10.4 [ml] and V90_Rectum_ = 1.1 ± 2.1 [ml] for *No-Spacer-Data* and *Spacer-Data* set, respectively. The patient specific distributions of V90_Rectum_ index presented here do not qualitatively differ from other usually investigated DVH points like V50_Rectum_, V70_Rectum_, which indicates that rectum is spared using spacer gel also in the spectrum of the lower doses. The daily distribution of V95_CTV_ index indicates that the application of OAR spacer gel does not affect the coverage of CTV for the TP constraints applied in this study. The MD of V95_CTV_ is 99.9 ± 2.5% and 99.8 ± 3.2% for *No-spacer-Data* and *Spacer-Data* set, and therefore fulfills the TP requirements in terms of daily target coverage.

As an example, the transversal and saggital views on dose distribution calculated on TP CT (“best case”) and daily dose distributions plotted over corresponding daily CT study (“worst case”), including the segmented contours are illustrated for patient *#2* of *No-Spacer-Data* set (Figure 1) and patient *#10* of *Spacer-Data* set (Figure 2). The selection criterion for these patients was the greatest difference between the value of V90_Rectum_ calculated for the TP CT and for daily CT study (indicated on Figure 1). As illustrated on the selected views on TP CTs ion beams allow for a highly conformal coverage of PTV (blue) and CTV (red) in the both patient cases. The application of the spacer gel (Figure 2, pink) increases the distance between the CTV (red) and rectum (brown) in such a way that the high dose fields do not reach the rectal frontal wall, as one can see comparing transversal CT slices on Figures 1b and 2b.

As expected, substantial improvement in rectum sparing is visible by comparison of daily dose distributions calculated for patient that had spacer gel implanted (Figure 1c,d) in comparison with patient without spacer gel implant (Figure 2c,d). The saggital view on patient #2 (no spacer gel, Figure 1d) clearly illustrates that, due to the rectum filling greater than on the TP CT (Figure 1b), part of the rectum shifts into the high dose field dedicated for the target volume (prostate) increasing the dose to the organ at risk. Comparison of the saggital views illustrated on Figure 2b and 2d shows that distancing prostate and rectum by application of spacer gel prevents deposition of the high dose field to the rectum. The partial high dose field deposited to the rectum is present in the region which has not been reached by the spacer gel (Figure 2d).

## Discussion

The presented dosimetric study quantifies the impact of application of spacer gel on rectum exposure to high doses during the ion therapy of PC. The treatment plans and daily dose distributions were evaluated and compared for 88 fully contoured TP and daily CT studies of two patient data sets (19 patients), with and without spacer gel. Presented analysis performed on the TP CT studies indicate a substantial reduction of maximal dose to rectum (Dmax-1ml_Rectum_) from 100.0 ± 1.0% to 90.2 ± 4.8%, when spacer gel is applied. The evaluation of daily dose distribution shows that daily rectal volume receiving dose higher than 90% of target dose (V90_Rectum_) could be reduced form tens to single milliliters, when spacer gel is applied. The presented results confirm and quantify our expectations from pre-clinical investigations on spacer gel described in [18], which showed that spacer gel has adequate physical properties for ion therapy (range of the ions in gel are predictable by TP system with unsubstantial error) and according to the previous experience from photon therapy [15,16], distancing prostate and rectum in ion therapy of PC helps to spare rectum from high doses.

Our result is in agreement with treatment planning studies performed by [17], who compared TP techniques like IMRT, rapid arc and Intensity Modulated Proton Therapy (IMPT) and reports dosimetric benefit for rectum from application of spacer gel for all these techniques. The simulations performed by [17] did not include day-to-day effects of prostate motion and patient positioning but focused only on the dosimetry of TP. In order to compare Weber’s results of rectum dosimetry given in relative numbers with outcome of this study given in the absolute rectal volume values, Weber’s average rectal volume receiving 50 Gy (V50 Gy, 64% of prescribed target dose) was divided by average rectal volume calculated over 8 patients analyzed by Weber. It was observed that V50 Gy index used by Weber decreases from 19% to 14% when spacer gel is applied, which corresponds to reduction of absolute rectal volume receiving 64% of prescribed dose from 11 to 9 ml assuming average delineated volume of rectum calculated over 8 patients of 65 ml (with spacer) and 57 ml (without spacer). The results of Weber are of the same order of magnitude as our finding: V70_Rectum_ decreases for TP CT studies from 12.2 ± 4.7 ml to 7.1 ± 2.2 ml, when spacer gel is applied. In another study [19] reports V60_Rectum_ for pencil beam scanning for a single CT data set to be in range 0-1% when spacer gel is applied, but does not report contoured rectal volume. Christodouleas et al. [19] suggests further dosimetric studies with a greater number of patient data to confirm the hypothesis of advantage from application of the anterior irradiation angles with spacer gel in presence of clinically applicable ion beam range verification methods.

According to recommendation stated in the “Report on the 4D treatment planning workshop 2013” [13] in order to guarantee the reliable comparison of the results of different research groups it is necessary to unify the evaluated parameters of treatment planning studies. In the discussed works comparison of the results is limited due to different TP parameters used for plan evaluation, patient to patient variations in the contoured regions of interest and different PTV concepts used for TP. More specifically, in order to compare the dosimetric results of TP studies on PC independent on the delineated rectal volume, on the basis of our experiences it is recommended for the similar TP and clinical studies in the future to use absolute volume of the rectum receiving certain dose level as a measure of rectal exposure.

First clinical results of application of spacer gel for radiation therapy show reduced hazard of rectal toxicity [28], which corresponds with the results of our dosimetric analysis. The reduction of daily rectal exposure by application of spacer gel allows for dose escalation or application of hypofractionated treatment protocol in ion beam therapy [10], as it was already proposed for photon therapy [28-31]. The results of our robustness analysis are clinically relevant dosimetric indication to define admissible fraction dose for PC treatment by means of rectal exposure and, therefore, to define the possible dose escalation or hypofractionation treatment protocol, taking into account the presence of prostate motion, patient positioning uncertainties and range variations of ion beam.

Pre-treatment imaging is a clinically applicable approximation and assumption of the patient anatomy at the moment of irradiation, even if a single CT study is an anatomy representation obtained before or after the treatment. Dosimetric analysis of target and rectal dose based on CT studies include effects related to soft tissue displacements in target region as well as range and re/positioning uncertainties and are present for both *No-Spacer-Data* and *Spacer-Data* set. The fundamental limitation of analysis performed in this study is the fact, that separation of these variables and quantifying their dosimetric effects in the patient is not applicable. The results of presented work based on the analysis of 88 CT studies demonstrate that the V90_Rectum_ index calculated over all cases decreases from 10.2 ± 10.4 [ml] for *No-Spacer-Data* to V90_Rectum_ = 1.1 ± 2.1 [ml] for *Spacer-Data* set. Assuming pre-treatment image guidance based on CT data, this result demonstrates the benefit of application of spacer gel in presence of soft tissue variations in the target region, even if random error of inaccurate patient re/positioning and beam range variations is included.

The outcomes of the robustness analysis presented in this work are based on the dose distribution calculations performed with TRiP98 treatment planning system which provides equal results to the clinically applied Siemens Syngo RT (HIT-TPS), Treatment Planning System (TPS) which is commercially used at HIT. Both TPS use the same experimental input data [25]. Richter compared dose distributions calculated with HIT-TPS and with TRiP98 software package and reports that the mean differences between dose distributions calculated by HIT-TPS and TRiP equal zero (STD below 1%) [32]. In the clinical routine, each TP calculated with HIT-TPS is experimentally verified by dose measurements performed in water phantom as it was proposed by Karger et al. [33] and reported by Henker et al. [34]. The comparison of TRiP98 and HIT-TPS and experimental verification of patient data routinely performed for HIT-TPS show clinical relevance of our simulations performed with TRiP98 software package.

Patient to patient variability of presented results demonstrates that the benefit from application of spacer gel is patient specific. The application of spacer gel protects rectum from the high dose, if prostate moves during the therapy course into the irradiation field. Further, Pinkawa et al. [35] reports that the application of spacer gel reduces larger prostate displacements and the distance between prostate and rectum separated by the spacer gel remains stable during the therapy course.

The human factor like quality of image registration and contour segmentation process which are part of the clinical routine were not evaluated in this study. The contouring process might have influence on the evaluation of dose parameters because volume and shape of the segmented contours might vary from observer to observer [36]. The impact of spacer gel application on daily bladder exposure was not evaluated in this work and should be a goal of further analysis.

## Conclusions

The application of spacer gel enlarges the distance between prostate and one of the most critical organs at risk, rectum, reducing rectal exposure to the high doses and, therefore, hazard of rectal toxicity during ion therapy of PC. The presented quantification provides results which could be used as a clinical indication for modification of PC radiotherapy protocol by dose escalation or hypofractionation.

## Additional file

Additional file 1:
**Analysis of HU stability of** ***No-Spacer-Data*** **set.**

## Abbreviations

HIT: Heidelberg Ion Beam Therapy Centre
PC: Prostate cancer
CT: Computed Tomography
TCP: Tumour Control Probability
IMRT: Intensity Modulated Radiation Therapy
IGRT: Image Guided Radiation Therapy
MRI: Magneto Resonance Imaging
TP: Treatment Planning
IPI: Ion Prostate Irradiation
DKFZ: German Cancer Research Center
HU: Hounsfield Unit
ROI: Regions of Interest
QA: Quality Assurance
PET: Positron Emission Tomography
CTV: Clinical Target Volume
PTV: Planning Target Volume
AP: Anterior-posterior
SI: Superior-inferior
LR: Left-right
TRiP98: TReatment planning for Particles
RBE: Radio Biological Effectiveness
3D: Three-dimensional
DVH: Dose volume histograms
MD: Median value
STD: Standard deviation
IMPT: Intensity Modulated Proton Therapy
TPS: Treatment Planning System
