# Supplementary material for: Ion therapy of prostate cancer: daily rectal dose reduction by application of spacer gel
Source: Radiat Oncol. 2015 Feb 27;10:56. doi: 10.1186/s13014-015-0348-1 (PMC4399750; doi:10.1186/s13014-015-0348-1)
Supplement: Additional file 1: — Analysis of HU stability of No-Spacer-Data set. [file 13014_2015_348_MOESM1_ESM.docx]

**Additional file 1**

Analysis of HU stability of No-Spacer-Data set.

Since the density of organs imaged on CT studies does not change on a daily basis, it is expected, that the distribution of HU values within one contour delineated on the CT study should not differ with regard to the same contour delineated on the reference CT study more than allowed for QA procedure limits (±10 HU for soft tissue and ±30 HU for bony anatomy). In order to test HU stability, three categories of volumes of interest (VOI) were considered: GTV, femur bones, entire CT study. VOIs were delineated on each CT study for all the patients. For each distribution of HU values collected within a contour a Gauss function was fitted and the daily-peak-position as well as daily-standard-deviation was calculated. An average of daily-peak-positions and an average of daily-standard- deviation are illustrated on Figure S4a and S4b respectively and are considered as a measure of day-to-day variation of HU values between CT studies for each of the patients. On both figures the standard deviation of daily- peak-position and daily-standard-deviation values is illustrated by the whiskers and demonstrates the variation of HU values between CT studies. The patient to patient variation of average daily-peak-position and average daily-standard-deviation confirms that the structure of delineated organs (distribution of HU values within a contour) vary from patient to patient, as the structure of organs vary between humans. The whiskers visible on the Figure S4 indicate that daily-peak-position and daily-standard-deviation (the position and shape of Gaussian fit) does not change for one patient between CT acquisitions. The double standard deviation of daily- peak-positions (2σ range) calculated within one patient for multiple CT studies (whiskers extend) reached maximally: 13.6 HU (patient 7) for GTV based histogram, 21 HU (patient 7) for femur bones based histogram, and 10.2 HU (patient 4) for histogram based on entire CT study. The standard deviation of daily-peak-position is of the same magnitude as the admissible variation for quality assurance of HU Look up Tables (HLUT) ±10 and ±30 for soft and bone tissue, respectively, which indicates that no significant changes in the quality of CT study occur in this test. The maximal standard deviation of all daily-standard-deviations (28 HU) indicates that the form of fitted Gauss function and, therefore, the daily distribution of HU values for all analysed contours do not change substantially on a daily basis. On the basis of presented analysis performed for No-Spacer-Data it was guaranteed that the quality of CT images used for robustness analysis presented in this work is stable in terms of HU values and allows accurate CT based dose distribution calculations.

**Figure S4** Stability of HU values for patients of No-Spacer-Data set. For each patient average daily-peak-position (a) as well as average daily-standard-deviation (b) were estimated from Gaussian fit of HU values distribution estimated from three categories of VOIs (entire CT, Femurs, GTV). The whiskers illustrate variation (standard deviation) of daily-peak-position and daily-standard-deviation calculated between CT studies.
